# Editorial: Recent advances with orthostatic intolerance/tachycardia in children and adolescents: international perspectives

**DOI:** 10.3389/fped.2025.1673874

**Published:** 2025-08-14

**Authors:** Amar Taksande

**Affiliations:** Department of Pediatrics, Datta Meghe Institute of Higher Education and Research, Wardha, India

**Keywords:** headache, tachycardia, children, orthostatic, postural

**Editorial on the Research Topic**
Recent advances with orthostatic intolerance/tachycardia in children and adolescents: international perspectives

Orthostatic intolerance (OI) and postural orthostatic tachycardia syndrome (POTS) are increasingly recognized as important contributors to functional disability in children and adolescents. These conditions are characterized by exaggerated cardiovascular responses to upright posture, leading to symptoms such as fatigue, dizziness, palpitations, visual disturbances, and sometimes syncope. Despite growing awareness, POTS often remains underdiagnosed or misinterpreted due to its heterogeneous presentation and limited pediatric-specific diagnostic frameworks. This Research Topic brings together recent global contributions that have advanced our understanding of pediatric OI, especially POTS, across several domains—from pathophysiology and diagnosis to management and patient experience. Zou R et al. (1) provided important epidemiological data from a large retrospective analysis of children presenting with unexplained headache or dizziness. Among nearly 2,800 patients, over 5% were diagnosed with POTS. His analysis revealed that female sex, older age, and low BMI were associated with increased likelihood of orthostatic intolerance—findings that may aid early recognition in clinical practice. Kakavand B et al. (2) contributed a valuable retrospective study on the psychological comorbidities seen in adolescents with POTS. His data revealed that 74% of affected children reported moderate-to-severe anxiety and/or depression. Notably, several patients experienced improvement in psychological symptoms following POTS-targeted therapy, even without psychiatric intervention—indicating a potentially bidirectional relationship between autonomic and emotional dysregulation. Morrow A et al. (3) explored the emerging association between long COVID and autonomic dysfunction. In her study of children with post-COVID symptoms, a substantial proportion met diagnostic criteria for OI and POTS. Using a 10-minute passive standing test, Allison's team documented a 71% rate of orthostatic abnormalities, underscoring the need for post-viral dysautonomia to be included in the differential diagnosis after COVID-19 infection. Boris J et al. (4) offered insights that challenge the rigid use of heart rate thresholds in diagnosing POTS. His study showed that adolescents with a peak upright heart rate below 100 bpm still reported symptom severity similar to those who met conventional tachycardia criteria. This finding supports the argument for a more symptom-centered, rather than number-centered, approach to diagnosis in pediatric POTS. Stewart J et al. (5), in a comparative study of diagnostic methods, evaluated the sensitivity and specificity of active standing vs. head-up tilt-table testing in adolescents. His findings showed that tilt testing remained the superior method for accurately identifying POTS, though standing tests may still hold value as an accessible screening tool, especially in primary care settings. Xu Bo et al. (6) introduced the use of the acceleration index, an electrocardiogram-derived parameter, as a predictor of therapeutic response to orthostatic training. This non-invasive tool demonstrated strong sensitivity and specificity in identifying children who are likely to benefit from structured reconditioning programs—providing a practical and accessible means of guiding treatment decisions. Bayrak Y et al. (7) and colleagues investigated the role of mast cell activation (MCA) in children and adolescents with POTS. Their prospective study identified elevated serum tryptase levels in patients with MCA-related symptoms, suggesting that immunologic pathways, including mast cell activation, may contribute to the pathogenesis of pediatric POTS. This expands the understanding of POTS beyond autonomic dysfunction alone, highlighting the relevance of immune-mediated mechanisms. Li J et al. (8), in a concise clinical review, emphasized the importance of non-pharmacologic approaches such as increased fluid and salt intake, regular exercise, and healthy sleep patterns. Importantly, the review also pointed to the need for early identification of comorbidities—such as hypermobility syndromes and anxiety—that may influence disease course and response to therapy.

Huynh P et al. (9) conducted a systematic clinical review examining the available pharmacological treatments for pediatric POTS. Although no FDA-approved therapies exist for this age group, his analysis suggested that medications such as ivabradine, midodrine, beta-blockers, and fludrocortisone may provide symptom relief in selected cases. However, authors also noted that pediatric treatment decisions are often extrapolated from adult data, and called for dedicated pediatric trials. Taken together, these studies highlight the growing consensus that pediatric POTS is a multi-system condition with autonomic, immunologic, psychological, and possibly genetic components. They also reflect an international effort to refine the diagnostic process, develop predictive tools, and personalize therapy. Importantly, the need for multidisciplinary care is emphasized, spanning pediatric cardiology, neurology, immunology, mental health, and education systems.

The psychosocial impact of OI in youth is another recurring theme. Adolescents with POTS often experience stigma, delayed diagnosis, and school disruption. Innovative solutions, such as school-based reconditioning programs and family-centered education, show promise in addressing these challenges and improving quality of life. Looking ahead, several research directions are clear. Large-scale longitudinal studies incorporating biological and psychosocial data are needed to better understand the disease trajectory. The development of pediatric-specific diagnostic criteria and evidence-based treatment guidelines remains a top priority. Additionally, wearable digital technologies and machine learning tools may enable earlier recognition and real-time symptom tracking. In conclusion, this Research Topic reflects the international momentum toward improving care for children and adolescents with orthostatic intolerance. The contributions of authors collectively offer new perspectives and practical solutions to a complex and often misunderstood condition. Their work fosters a shared vision: to ensure that every child with OI or POTS receives timely recognition, individualized care, and hope for recovery.
